# Topics of Public Interest

**Published:** 1905-09

**Authors:** 


### TOPICS OF PUBLIC INTEREST.

### Yellow Fever Immunity.

DR. DOTY THINKS IT USUALLY COMES FROM DISEASE ITSELF.

DR. A. H. DOTY, health officer of the port of New York, when discussing the much debated question of immunity from yellow fever recently, said that there was a difference of opinion on the subject, even among experts. He pointed out that permanent immunity among natives in yellow fever areas came rather from their being attacked by the fever in infancy than from heredity. He said also that the discovery of malarial fever organisms by the French army surgeon, Laveran, was exceedingly valuable in establishing the differential diagnosis between malarial and yellow fever. He said:

Some difference of opinion exists, even among experts, as to the immunity or susceptibility of persons exposed to yellow fever. There is a popular and widespread belief that those who are born or who have lived for a long period where yellow fever is more or less constantly present—that is endemic—are immune to this disease. While this is apparently so, the fact is that neither of these conditions can be depended on to confer immunity, although they diminish the susceptibility to an attack of yellow fever.

When permanent immunity exists among the natives in sections where yellow fever is epidemic, it is not so apt to be due to what is regarded as hereditary immunity as to the protection afforded by an attack of yellow fever in infancy or childhood. At this period the disease is very mild and often unrecognised, and frequently occurs in endemic areas. Furthermore, it has been conclusively shown that many natives, as well as those who have lived for a long period where yellow fever is endemic, and who believed they were immune, have contracted the disease.

It is commonly understood that the colored race enjoy immunity. This, however, is not a fact. They are susceptible to yellow fever, and contract it, but the mortality is very low. In other words, while they are not immune from the disease, as a rule, they have it in a mild form. There are also some persons who seem to have a natural immunity, not only to yellow fever, but to other infectious diseases, as they apparently remain with impunity in infected areas.

There is no doubt that this is often due to an attack of infectious disease in an unrecognised form or in very early life. However, these persons also contract infectious disease, even after they have been many times in close proximity to it. So far as yellow fever is concerned it is reasonable to assume that the only permanent immunity is obtained by an attack of the disease.

Instances are cited by almost all authorities where persons have suffered from a second attack; still, when these cases are carefully analysed, it is found that both attacks only in rare instances came under the observation of the same physician, or whoever reported the case. Therefore, the evidence of the occurrence of a previous attack depends largely upon the statement of the person affected. This cannot be accepted as satisfactory scientific evidence; furthermore, dengue, a disease which is well known in the South, and also some forms of malarial fever, sometimes bear a striking resemblance to yellow fever. Nothing is more certain than that in the past and even at the present time these diseases have been mistaken for yellow fever.

We now have an exceedingly valuable aid in establishing the differential diagnosis between yellow fever and malarial fever. In 1880 Dr. Laveran, a French army surgeon, stationed in Algeria, discovered the organism which is the cause of malarial fever. The value of this cannot be overestimated, inasmuch as it places in our hands means by which the presence of malarial fever in many cases can positively be detected.

Although bacteriological research has thus far been unable to determine by examination of the blood or otherwise the presence of yellow fever, the examination of the blood of those who are held in suspicion may show the presence of the malarial organism, and therefore explain the elevation of temperature, and the like, and practically aid in excluding the presence of yellow fever.

It is for the reason, therefore, of ascertaining if persons with high temperatures or those who present suspicious symptoms are suffering from malarial fever that the examination is made of the blood of all suspects who are removed from incoming vessels at the New York quarantine station. In a number of instances cases which have been reported as yellow fever on vessels arriving at New York, particularly sailing vessels, where additional or secondary cases have occurred in transit, the examination of the blood has proven the presence of malarial fever.

As a résumé, it may be said that the immunity of natives residing in areas in which yellow fever is endemic is largely due to an attack of the disease in infancy or childhood. This view is supported by the fact that reliable evidence has been presented to show that natives who are believed to be immune sometimes contract yellow fever. Neither can a person who has lived in an endemic area for a number of years, or a person who is apparently immune to other infectious diseases be regarded as protected inasmuch as satisfactory proof has been presented to show that they have also contracted yellow fever. On the other hand, those who are natives and those who have lived in an endemic area for a long period are surely not so susceptible as those who come from sections of the world where the disease does not exist. For instance, a person going from the northern part of this country to a yellow fever area may be regarded as particularly susceptible to the disease.

There is little doubt that an attack of yellow fever furnishes practically permanent immunity to the person affected, and that the possibility of a second attack is exceedingly small, so much so, that it is not necessary to take it into consideration in establishing quarantine regulations, and from a practical standpoint it is fair to assume that if a person has yellow fever the report that the said person has had a previous attack of the disease is open to doubt.—*The Tribune,* August 16, 1905.

---

### "A University School for Nurses."

In the issue of this Journal for July, in the current year we published an address by Dr. F. Park Lewis, of Buffalo, delivered at the annual commencement exercises of the Brooks Memorial Hospital at Dunkirk. The *Buffalo Evening News* in a recent issue comments at length on the essential topic discussed by Dr. Lewis. We take pleasure in reprinting the *News* editorial in full:

### A UNIVERSITY SCHOOL FOR NURSES.

Something a little out of the ordinary course of sound personal advice and exhortation to high achievement in pursuit of noble ideals was presented to the graduating class of nurses at the Brooks Memorial Hospital at Dunkirk by Dr. F. Park Lewis of this city at the recent commencement. Dr. Lewis's address appears in full in the July issue of the Buffalo Medical Journal, and it has a timely bearing on an enterprise, which has enlisted the ardent support of the Journal as it has of the *News* and other members of the secular press—the creation of a real University of Buffalo.

Dr. Lewis's devotion to the most exacting requirements of one of the most exacting of the curative sciences, that dealing with the sight, is well known. His canvass of the requirements of the trained nurse of today in the address to the graduating class set a standard of thoroughness in training and of sharply defined knowledge in every branch of the art scarcely less necessary nowadays than the physician's own, that might prove discouraging to students if it were not that in some of the greatest of the hospitals in this country means and systems of training have been supplied which make possible accomplishment of all that medical success as now measured may demand of the doctor's indispensable assistant. An outline was given of what, as an example, the great Johns Hopkins Hospital is doing to realise to the uttermost these demands on the trained nurse of today:

In her laborious hours of preparation she must master an unfamiliar nomenclature, she must conquer the difficulties of modern asepsis, she must school herself to stand by the surgeon without growing faint; she must learn to govern her tongue, to control her temper, to be quiet, to be ready, to be dexterous—to have the qualities of a soldier while retaining the heart and sensibilities of a woman. All this she must learn besides the technical points of the work she has undertaken. * * * The good dame who used to make flaxseed poultices and draughts of sage tea could give no adequate assistance to the physician or surgeon of today. The modern trained nurse, as well as the physician, must understand that invisible pathogenic germs are more formidable than lions in the path and to do this she must have some conception of the principles of bacteriology. She must know the meaning of the zig-zag temperature looking like saw-teeth on the daily chart for general sepsis must have immediate recognition. She must be familiar with the details of the diet kitchen, for here is often a path where the physician fails to follow, and the directions which she receives are too frequently glittering generalities, the details of which she must herself supply.

These are but a few of the categorical requirements set forth by the speaker, and the point most strongly impressed in sequence to those was that in the many schools now imparting instruction along these general lines—there are eleven in Buffalo alone, exclusive of the State Hospital—there is no uniformity of curriculum or of methods of study. In the state of New York there were in 1903, 13,779 pupil nurses. In Greater New York there are almost as many trained nurses enrolled as there were five years ago in the entire United States. Yet the instruction of all these is to an extent haphazard—dependent on the efforts of busy practitioners who can ill afford the time or the distraction from their regular work called for by lectures to the classes at hospitals. The faults of the present system or lack of it hardly need to be enlarged upon. That the profession of nursing makes progress in spite of the handicap of scattered efforts at instruction is something to be thankful for, but the condition may be avoided, and the example already mentioned points to a better way here as well as elsewhere in this state.

The hospital school at John Hopkins University, Baltimore, is part of the Johns Hopkins Hospital. The school buildings are separated from the hospital buildings, however, and the instruction in the properties and preparation of foods and in their application to the needs of the sick is given in a model kitchen equipped for teaching purposes.

The instruction for the first six months is given to a study of household economics, hygiene and sanitation, anatomy, physiology, materia medica and elementary nursing. This is considered a probationary period, and is designed to give the student some of the fundamental principles of nursing before she is brought into contact with patients. Those who give satisfactory evidence of a general fitness for the work of nursing are then placed in the wards of the hospital for further work and study.

Teaching is given in the operating rooms and other clinics as well as at the school. The hospital has 360 beds, so that a large practical experience is afforded the student nurses. The faculty is large, there being four assistant superintendents, two instructors in practical nursing, two in dietetics, one instructor in massage, ten head nurses and assistant instructors (all of them women) besides the physicians.

During the first year students are instructed in marketing, the cost and care of foods as well as cooking; the care of kitchens, pantries, utensils, fuel and the like, as well as especial training in the preparation of food for infants and for the sick. A study of plumbing and drainage, the principles involved and care necessary to maintain healthful conditions is included, and also a careful study of laundries and laundry work and of linen room and the care and distribution of linen, as well as of bedrooms and surgical wards. The three years' course required in the Johns Hopkins school makes possible all this preliminary work, and it gives to its graduate nurses a training almost equal to that of the Drexel Institute in Domestic Science, the Boston Cooking School and the Philadelphia School of Massage in their respective branches.

To the suggestion that this establishes a high standard which many pupils might not reach, Dr. Lewis says "set the standard as high as possible, but if necessary have two grades of nurses," to do different kinds of work, of course, and necessarily to command different rates of pay. He then went on to outline a plan which, he said, had kindled his imagination, and which briefly is the establishment of a great central school of nursing as part of the to-be complete University of Buffalo. This he proposes to bring about by coöperation of all the schools now engaged in training nurses in Buffalo, and in like manner he would centralise the work throughout the state. The details of such coöperation, only hinted at in broad terms in the doctor's address, will suggest themselves. The keynote of what he would bring about has been given. The advantages of such a centralised and greatly enlarged training and of a diploma representing it are obvious. As a contribution to the movement for an enlarged university Dr. Lewis's very practical suggestion must certainly command attention. Dr. Potter, editor of the MEDICAL JOURNAL, evidently holds it as important, from the prominence given it in the JOURNAL. A response from the various schools is naturally expected, and it seems hardly probable that it will fail to help along the good work in the interest of university development so happily begun.

The enlightened view of the *News* is to be commended and Mr. Edward H. Butler, the editor and proprietor, is to be congratulated upon his administration of such a progressive newspaper.

### "An Act Prohibiting Advertisements for the Cure of Sexual Diseases."

It is extremely gratifying to learn that there is at least one state in the union with the wisdom and firmness to grasp the problem suggested by the above heading, in a way that can not fail to achieve a reform which we wish might become general throughout the country.

The legislature of the state of Washington, and the organised body of the profession of that state, are to be congratulated upon having secured the passage through both houses of this most commendable bill, the title of which we quote above, against the opposition of the newspapers and quack doctors of the state. It prohibits the publication or printing of any advertisement to cure genitourinary diseases, to restore lost manhood, or to treat this class of disorders. The penalty is imprisonment from one to six months for the editor or owner of any paper or proprietor of any printing establishment who publishes such advertisements.

*Northwest Medicine* (March, 1905,) states in an editorial note, referring to this act, that the governor is expected to sign this bill and that results may soon be expected from it.

Is it possible to conceive of the clearing of the atmosphere which will follow as the result of the enforcement of such a law? In the face of these recorded facts, no one can deny that we still have something to learn from the self-respecting action of our sister state in the far northwest.

Could such a law be made national in character, it would strike at the very roots of an evil as glaring as it is loathsome, and would, without question, have a wide influence in lessening the number of juvenile offenders and moral degenerates which abound throughout the country, while adding immeasurably to the dignity and self-respect of our lay press.—*Cleveland Med. Journal,* April.

---

Systematic Examination of the Eyes of Defectives.—F. Park Lewis (*American Medicine,* Aug. 5, 1905,) shows that the nutritive, psychic and mental processes are all unfavorably modified by eyestrain. In view of this fact he believes every dull and nervous child, every person with incipient tuberculosis, every delinquent child, every applicant for admission to state hospitals, every epileptic with reasonable intelligence is entitled to examination of eyes for relief of this form of nervous waste if defect is present.

